# Examining the effects of mental health literacy on stigma: study of Zimbabwe Prisons and Correctional Service (ZPCS)

**DOI:** 10.1192/bji.2025.11

**Published:** 2025-11

**Authors:** Darlington Chiobvu, Hauwa Onifade, Gwatirera Javangwe, Musa Sami

**Affiliations:** 1 Academic Unit of Mental Health and Clinical Neuroscience, University of Nottingham, Nottingham, UK; 2 Department of Applied Psychology, University of Zimbabwe, Harare, Zimbabwe; 3 Institute of Mental Health, Nottingham, UK

**Keywords:** Mental health literacy, mental health stigma, prison, correctional service, Zimbabwe

## Abstract

**Background:**

Mental illness affects an estimated 500 million people globally, with 85% living in low- and medium-income countries (LMICs). Research has shown that people with mental illness are over-represented in the criminal justice system compared with the general population. There is limited information available on institutional attitudes towards mental illness in LMICs.

**Aims:**

This study aimed to examine mental health literacy (MHL) and mental health stigma (MHS) among Zimbabwe Prisons and Correctional Service (ZPCS) officers.

**Method:**

A cross-sectional study using an online survey was conducted among ZPCS officers (*N* = 163) between August and December 2022. Data were collected using the Mental Health Literacy Scale (MHLS) and Attitudes Towards Mentally Ill Offenders (ATMIO). The primary hypothesis was that increased MHL is inversely related to MHS in this group.

**Results:**

A significant inverse correlation was found between MHL and MHS (*r* = 0.36, *P* < 0.001). A regression analysis controlling for age and sex showed that MHLS is a statistically significant component in this model, indicating that MHL is associated with reduced MHS (*P* < 0.001).

**Conclusions:**

In this group, increased MHL is associated with decreased MHS. This suggests that interventions to increase MHL should be further evaluated in these settings.

## Mental health literacy in developing countries

Mental health literacy (MHL) is essential for mental health development, especially in low- and middle-income countries (LMICs) where there are limited mental health resources,^1^ such as trained mental health staff and inadequate funding.^2^ According to research, MHL theory stems from the health literacy concept.^3^ Health literacy is described as the capacity to read, comprehend and apply health information to make informed healthcare decisions and follow instructions recommended for treatment.^4^ From this concept, Jorm et al examined MHL among the general public’s attitude towards mental health disorders and existing treatment and found that few participants were able to correctly recognise depression (39%) and schizophrenia (27%) in a vignette.^5^ Based on these findings, the authors defined MHL as ‘knowledge about mental health disorders that are associated with their recognition, management, and prevention’.^6^

It is estimated that 500 million people worldwide are affected by mental illness,^7^ and 85% of these reside in LMICs.^8^ Studies have argued that mental illness recognition and treatment rates are significantly lower in LMICs;^9^ for example, a cross-sectional survey conducted among 250 healthcare workers in Zambia and South Africa found moderate levels for MHL, ranging from low to high. Participants with a higher level of education demonstrated a remarkable ability to recognise mental health disorders. Additionally, those who had experience of using cognitive health-related assessment tools had higher MHL scores.^10^

Furthermore, a group of studies has shown that a lack of MHL has resulted in people being likely to believe in supernatural beliefs in some parts of LMICs. For example, a review in Nigeria explored knowledge, perceptions and attitudes towards mental health issues. The study found that mental health disorders were perceived as supernatural, such as the possession of evil spirits, witchcraft and divine punishment.^11^ Another study conducted in Zambia, which explored cultural beliefs surrounding mental illness, discovered that traditional beliefs such as being bewitched, possessed by demons and having sex with uncleansed widows were deeply embedded in thoughts and perceptions as the causes of mental illness.^12^ Similarly, a study in Zimbabwe found that 30 individuals with mental illness and who were interviewed believed their illness stemmed from witchcraft. The findings also revealed that these individuals with mental illness, and 18 participants from the community who participated in three focus-group discussions, attributed the causes of mental illness to spirituality, ancestral spirits and being possessed by evil spirits.^13^ Another study conducted in Zimbabwe, which explored the association of common mental disorders (CMDs) among primary care attenders, found that CMDs were caused by being bewitched and ‘thinking too much’.^14^

## MHS in developing countries

The stigma associated with mental illness (MHS) has resulted in a severe problem that affects patients, their loved ones, institutions and healthcare professionals who work closely with people with mental health difficulties.^15^ The American Psychological Association defined stigma as ‘the negative social attitude attached to an individual’s characteristic that may be regarded as a mental, physical, or social deficiency’.^16^ According to the Mental Illness Stigma Framework, stigma associated with mental illness is characterised by four subtypes: internalised, experienced, anticipated and perceived.^8^

In most African countries, negative preconceptions about mental illness have created fear of individuals with mental illness due to a lack of understanding of mental disorders and effective policies that address the condition.^17^ As a result, this could lead to an increased risk of human rights violations of people with mental illnesses, reduced access to health services and delayed help-seeking behaviour.^8^ For example, a systematic review that looked at the barriers and facilitators of treatment-seeking behaviours in LMICs for depression, epilepsy and schizophrenia discovered that knowledge deficits, beliefs and stigma were all barriers to treatment seeking across disorders.^18^ Furthermore, it was noted that some Arab countries held stigmatising attitudes toward mental illness, and people with mental health disorders faced discrimination and poverty.^19^ As such, many people do not seek treatment from mental health services in the initial stages; instead, they are examined by religious or spiritual healers whose task is to free the patient from ‘evil’.^20^

## MHL and stigma in the criminal justice system

### MHL in the criminal justice system

Previous studies have indicated that people with mental illness are over-represented within the criminal justice system (CJS) compared with the public.^21–24^ This over-representation has been a growing concern for healthcare providers, policymakers, correctional officers, human rights advocates and lawyers.^25,26^

Studies have claimed that MHL within the CJS is under-researched.^22,27^ Among those studies that examined MHL among correctional officers, one assessed their mental health knowledge and work-related self-efficacy (*N* = 40) using the Multiple-Choice Knowledge of Mental Illness Test. The study found that correctional officers had lower levels of mental illness knowledge, but an elevated level of self-efficacy, when working with people in prison with mental illness. Officers with higher education qualifications reported higher MHL than those with lower qualifications.^28^

### MHS in the criminal justice system

Stigma towards people with mental illness is considered to be high among correctional officers in developed countries. Research suggests that this may, in part, be due to media coverage reports portraying individuals with mental illness as violent.^24^ In the USA, one study exploring stigma perceptions among judges and attorneys discovered that judges and prosecutors had negative attitudes toward mental illness. As such, they viewed such people who had encountered the CJS as a greater risk to the community.^29^ Similarly, another study found that probation officers rated people in prison with mental illness as dangerous compared with those without mental illness.^30^

Furthermore, one study in the USA found that older custodial staff had more negative attitudes toward people with mental illness.^31^ Some studies have argued that these stigmatising attitudes may result in discrimination against people in prison with mental illness, being restricted in accessing treatment and leading to punitive measures. As a result, this could have a negative impact on the well-being and treatment outcomes of people with mental health difficulties.^31^ In contrast, other studies found neutral and positive attitudes toward mental illness by probation, parole and custodial officers.^32,33^ Others found that daily contact with people in prison diagnosed with mental illness was associated with positive attitudes compared with monthly contact.^34^

### Training of correctional officers

There is evidence that mental health training modalities, such as Crisis Intervention Teams (CITs) and Mental Health First Aid (MHFA), can improve correctional officers’ attitudes towards mental illness.^30^ MHFA was developed in Australia in 2001 and is defined as ‘the help provided to a person developing a mental health problem or in a mental health crisis’.^35^ MHFA is a training programme for anyone with limited or no experience and who desires to identify mental health issues and provide early assistance to people who want emotional or mental healthcare.^35^ One study examined the effectiveness of MHFA among 105 jail staff in the USA who completed the MHFA training, and reported that it was beneficial for both their employment and everyday life.^36^

Furthermore, the CIT was developed in the USA in 1998 by a police officer in response to the events following the fatal shooting of a man who had wielded a knife and with a history of mental health and substance use.^37^ Since then, the CIT has been proposed as a promising intervention model to address certain challenges faced by correctional officers when interacting with people experiencing mental illness symptoms.^38^ One study examined the perceptions and preparedness of correctional officers working with people incarcerated with mental illness and in crisis. Among participants, some correctional officers were certified in CITs while others were not.

That study found that the two groups showed no differences in perceptions. However, those correctional officers who were certified in CITs reported feeling more equipped to engage with people in prison experiencing mental health symptoms and those in crisis. This group of CIT-certified correctional officers also demonstrated more positive attitudes toward these individuals.^39^

### Forensic mental health services in Zimbabwe

The World Health Organization’s Special Initiative for Mental Health Assessment (2020) highlighted that forensic mental health services in Zimbabwe house a significant portion of the country’s mental health patients. For example, the Zimbabwe Prisons and Correctional Service (ZPCS) operates two special forensic institutions in Harare and Bulawayo, at Chikurubi and Mlondolozi maximum-security prisons, respectively. The Zimbabwe Mental Health Act (MHA) of 1996 (Section 107) governs these facilities for the assessment and treatment of both males and females with mental illness and who are detained in these special forensic institutions. The MHA (Statutory Instruments 15, Section 107 of 1996a), the Mental Health Regulations Statutory Instrument 62 of 1999, the Mental Health Policy of 2004 and the Prisons Act (chapter 7:11) are the key legislative frameworks that guide these special institutions.^40^ Approximately 478 individuals with mental illness (not convicted) were admitted at Chikurubi Special Institution (Northern region) against a holding capacity of 150, resulting in severe overcrowding. Meanwhile, at Mlondolozi (Southern region) it was reported that 301 patients were admitted against a holding capacity of 406.^41^

### The present study

There is limited research conducted in LMICs, such as Zimbabwe, that have explored MHL and MHS in regard to mental illness. Most research in Zimbabwe has explored rehabilitation interventions to reduce inmate reoffending,^42–44^ but this does not tell us about the level of care provided by the services themselves. Therefore, to address this gap, our study aimed to examine MHL and MHS in the ZPCS in regard to mental illness. Our first task was to determine whether MHL is related to MHS among this group; if such a relationship is demonstrated, increasing MHL may be a means of decreasing MHS. As a result, our primary hypothesis was that increased MHL (as measured by the Mental Health Literacy Scale (MHLS), in which a higher score indicates increased literacy) would be inversely related to MHS in this group (as measured by Attitudes Towards Mentally Ill Offenders (ATMIO)), where a higher score indicates low stigma). The following hypotheses were exploratory: officers with more experience are likely to show a positive attitude towards mental illness (H1); officers who have daily contact in prison with people with mental illness are likely to show positive attitudes and knowledge about the condition (H2); and officers who have received mental health awareness training are likely to (a) show a positive attitude towards people in prison with mental illness and (b) have higher levels of MHL (H3).

## Method

### Study design and population

A cross-sectional study using an online survey was conducted between August and December 2022. The survey featured participants from all ten provinces, for 163 ZPCS officers (see Table 1). A 65-item questionnaire, consisting of 3 major sections, was used to measure the key constructs. The survey included one demographic section and two standardised scales, MHLS^45^ and ATMIO.^46^ Both survey questionnaires were in English and were not translated into vernacular languages, because English language is a requirement entry during the recruitment process for ZPCS officers.

**Table 1:** Numbers of participants in this study, by province in Zimbabwe

Province	Number of participants
Harare Metropolitan	50
Bulawayo Metropolitan	11
Midlands	22
Manicaland	26
Masvingo	15
Mashonaland East	4
Mashonaland West	5
Matabeleland North	5
Mashonaland Central	11
Matabeleland South	14

The MHLS has 35 items and 7 attributes: knowledge of seeking information, risk factors, recognised disorders as causes of mental illness, self-treatment, professional help available and attitudes that promote recognition or application of help-seeking behaviours.^6^ The first 15 items are scored on a 4-point Likert scale while items 16–27 are scored on a 5-point Likert scale, with items 20–28 scored in reverse. The last section of the questionnaire, items 29–35, is scored on a 5-point Likert scale. For reliability, McDonald’s omega and Cronbach’s alpha values were 0.797 and 0.789, respectively.^47^

ATMIO is a 31-item questionnaire developed by Church et al^46^ to elicit both general and specific attitudes toward people in prison with mental illness. It is graded on a 6-point Likert scale ranging from ‘disagree strongly = 1’ to ‘agree strongly = 6’. The instrument has been factored into four subcategories in addition to the overall attitudinal score: harmful stereotypes, diminished responsibility, community risk and rehabilitation and compassion. The items are summed to determine the overall attitudinal score, with 13 of these reverse scored. Higher scores indicate a more tolerant attitude toward people in prison with mental illness. Initial ATMIO reliability was *a* = 0.73 when it was under development,^48^ later rising to 0.88.^46^

### Sampling strategy

A non-probability convenience sampling method and a cross-sectional survey design were employed to obtain data from approximately 12 583 correctional officers.^39^ All eligible participants were recruited through social media (WhatsApp), and those interested were asked to complete the survey via a link distributed among ZPCS WhatsApp groups. Before taking part, participants were informed that the study was voluntary and they were provided with an online information sheet with the study’s aims and objectives. The inclusion criteria for participants were: any gender, 18 years old or older, employed and with a basic understanding of English. Those excluded were retired ZPCS officers.

### Sample size

Using G-Power 3.1 testing for a correlation of −0.3 with the alpha set at 0.05 and the beta set at 0.8 (i.e. 80% power), the minimum sample size required was 64 participants.

### Statistical analysis

The Statistical Package for Social Sciences (SPSS, version 28) was used to analyse the data at a significance level of 0.05. Descriptive statistics were analysed using percentage, mean and standard deviation. A correlation (Pearson’s *R*) was used to determine the relationship between MHS (measured by ATMIO) and MHL (measured by MHLS). Also, a regression analysis was used to examine the link between MHLS and ATMIO when age and sex were considered. Furthermore, an independent-samples *t*-test was used to compare the means and standard deviations of how often participants were in contact (daily *v*. not daily) with offenders with mental illness, and who attended and did not attend any mental health awareness training programmes.

### Ethical considerations

The authors assert that all procedures contributing to this study complied with the ethical standards set by the relevant national and institutional committees on human experimentation, and comply with the guidelines of the Helsinki Declaration of 1975 as amended in 2013.^49^ Ethics were approved by the Faculty of Medicine & Health Sciences Research Ethics Committee at the University of Nottingham (approval no. FMHS 507-0322) and the Medical Research Council of Zimbabwe (MRCZ) (approval no. MRCZ/A/2923). Additionally, the Commissioner General’s Office of Department of Research and Development team granted permission to conduct the study within ZPCS facilities with its officers. Participants were provided with online informed consent and debriefing information after completing the survey.

## Results

### Participants’ characteristics

A total of 163 ZPCS officers participated in this study between August and December 2022. Among participants were both senior- and junior-ranking officers (*N* = 117, 71.8%) and mental health professionals, all of whom are employed by ZPCS as correctional officers, including psychiatrists (*n* = 1, 0,6%), psychologists (*n* = 4, 2.5%), social workers (*n* = 9, 5.5%), psychiatric nurses (*n* = 9, 5.5%), participants who preferred not to disclose their profession (*n* = 6, 3.7%) and those in the ‘other’ category (*n =*18, 11%). As shown in Table 2, the majority of our sample were males (62%) and the minority females (38%). The mean age of participants was 39.4 years (s.d. = 8.17), and the average mean for number of years served in the organisation was 15.65 (s.d. = 8.18).

**Table 2:** Participants’ demographic characteristics (*N* = 163; 158)

Characteristics	Mean	s.d.	Range	Frequency	Validity, %
Age (years)	39.41	8.17	21–58		
Gender					
Male	–	–	–	100	62.1
Female	–	–	–	63	37.9
Other/prefer not to say	–	–	–	–	–
Number of years served	15.65	8.18	0–36	–	–
MHLS score	114.17	14.96	80–150	–	–
ATMIO score	101.45	11.62	73–133	–	–

MHLS, Mental Health Literacy Scale; ATMIO, Attitudes Towards Mentally Ill Offenders.

### Primary hypothesis

**Fig. 1:**
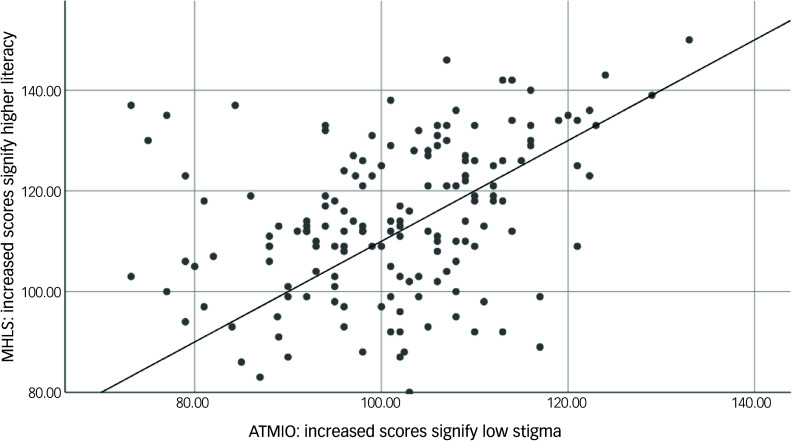
Scatter plot of MHLS versus ATMIO. MHLS, Mental Health Literacy Scale; ATMIO, Attitudes Towards Mentally Ill Offenders.

A Pearson correlation coefficient was used to test the primary hypothesis to determine whether MHL is related to MHS among this group. The results show a positive correlation between high scores for MHLS and ATMIO, indicating low stigma: *r* (158) = 0.36, *P* = <0.001. Figure 1 shows the direction of the relationship between MHLS and ATMIO scores.

Going beyond Pearson correlation, a regression analysis was conducted to evaluate the association of MHLS and ATMIO scores when sex and age were considered. The model was significant: *F* (3153) = 9.872, *P* < 0.001. The results revealed that MHLS remains a statistically significant factor in this model (*P* < 0.001); sex, however, is not a significant predictor in this model (Table 3), because *P* = 0.524. As such, this suggests that sex does not affect levels of MHS among this group. Regarding age, the results are statistically significant (*P* = 0.040), indicating that older age is associated with a lower MHS.

**Table 3a:** Model summary

Model	*R*	*R* ^2^	Adjusted *R* ^2^	s.e. of the estimate
1	0.403^a^	0.162	0.146	10.62953

a. Predictors: (constant), what is your age?

**Table 3b:** Analysis of var^a^

Model	ANOVA summary for regression predicting stigma score from age	Sum of squares	d.f.	Mean^2^	*F*	*P*
1	Regression	3346.147	3	1115.382	9.872	<0.001^b^
	Residual	17 286.988	153	112.987		
	Total	20 633.136	156			

ANOVA, analysis of variance.

a. Dependent variable, stigma score.

b. Predictors: (constant), what is your age?

**Table 3c:** Coefficients^a^

Model	Regression coefficient for predictors of ATMIO	Unstandardised beta	Coefficients, s.e.	Standardised coefficients, beta	*t*	*P*
1	(Constant)	64.261	8.673		7.409	<0.001
	MHLS	0.260	0.057	0.339	4.564	<0.001
	Sex	−1.200	1.879	−0.051	−0.639	0.524
	What is your age?	0.233	0.112	0.165	2.074	0.040

MHLS, Mental Health Literacy Scale; ATMIO, Attitudes Towards Mentally Ill Offenders.

a. Dependent variable, ATMIO.

### Secondary hypotheses

Hypothesis 1: officers with more experience are likely to show a positive attitude towards mental illness.

Pearson correlation coefficient was computed to assess the linear relationship between years of experience serving in the current job and ATMIO. There was a positive correlation between the two variables: *r* (158) = 0.184, *P* = 0.021, as shown in Table 3. Therefore, the results suggest a weak but significant association between having more experience and less stigma.

Hypothesis 2: officers who have daily contact with people in prison with mental illness are likely to show positive attitudes and knowledge about mental illness.

An independent sample test was conducted to compare how often participants were in contact (daily *v*. not daily) with people in prison with mental illness. The results indicated no statistically significant difference in scores for daily contact (*M* = 100.6, s.d. = 13.01) and not daily: *M* = 102, s.d. = 10.76; *t* (156) = −0.680, *P* = 0.497, with a small effect size (Cohen’s *d* = 0.112). These results suggest that having daily contact with people in prison with mental illness is not associated with any change in stigma.

Hypothesis 3: officers who received mental health awareness training are likely to (a) show a positive attitude towards people in prison with mental illness and (b) have higher levels of MHL.

A *t*-test analysis was conducted. For H3, the results from this analysis demonstrated that there was no significant difference in stigma score between persons who attended training (*M* = 102.60, s.d. = 13.23) and those who did not (*M* = 100, s.d. = 10.43; *t* (156) = 1.01, *P* = 0.337), with a small effect size (Cohen’s *d* = 0.164). There was a statistically significant difference in MHL scores between those who had received mental health training (*M* = 119.23, s.d. = 14.20) and those who had not (*M* = 110.96, s.d. = 14.58; *t* (161) = 3.65, *P* = 0.001), with moderate effect size (Cohen’s *d* = 0.583). As a result, those who had received mental health awareness training were considered knowledgeable about mental health.

## Discussion

This study aimed to investigate MHL and MHS of mental illness in the ZPCS. To date, there is limited research that has examined correctional officers’ MHL and MHS towards people in prison with mental illness in LMICs. However, several studies have explored MHL and the MHS of correctional officers in regard to mental illness, and those affected, in developed countries.^22,31,33^

The primary hypothesis was to determine whether MHL is related to MHS among this group. We hypothesised that increased MHL (as measured by MHLS, where a higher score indicates increased literacy) would be inversely related to MHS in this group (as measured by ATMIO, where a higher score indicates low stigma). Our hypothesis is consistent with previous research that examined the relationship between MHL levels and MHS.^50,51^ Additionally, after further analysis when sex and age were considered to evaluate the association of MHLS and ATMIO scores, this remained after adjustment. We found that sex was not a significant predictor in our model, suggesting that being male or female did not affect levels of MHS. Previous research found a significant difference in MHL between male and female participants. However, regarding age, the findings indicated no significant difference in MHL levels.^23^ In contrast, our results revealed a statistically substantial association between older age and low levels of MHS. This may be attributed to greater years of experience in this career, and resulted because age is related to years of experience in the post, making it impossible to separate the two (age and years).

We also hypothesised (H1) that officers with more experience are likely to show a positive attitude towards people in prison with mental illness. Our findings support this hypothesis, revealing a weak (*r* = 0.184) but significant relationship between having experience as a correctional officer and less stigma. Based on further analysis of our primary hypothesis, one might argue that our findings for H1 can be linked to age, implying that the older you are, the more experience you have in the job. Previous research revealed that daily interaction with people in prison with mental illness was more likely to result in positive attitudes than monthly contact.^34^ Our findings do not support our hypothesis (H2) that officers who have daily interaction with people in prison with mental illness are more likely to have favourable views and understanding regarding mental illness. There was no significant difference in ratings between daily and non-daily contact. We concluded that having regular contact with people in prison with mental illness had little effect on stigma. Our final hypothesis (H3) was that officers who had received mental health awareness training would be likely to show a positive attitude towards people in prison with mental illness. Previous research found mixed evidence that mental health training had improved stigma attitudes among correctional officers. Our research found no significant difference in stigma score between those who attended and those who did not participate in mental health training, but found a difference in MHL. Overall, these results are consistent with previous research that examined the perceptions and preparedness of correctional officers who were certified in CIT and those who were not.^39^

### Limitations

There are limitations to this study, which is in keeping with research with low-resource settings. First, the study used a cross-sectional survey, which precludes determination on causal outcomes. Second, the findings of this study are not generalisable because convenience sampling was used as the preferred sampling method. Therefore, there is a possibility of bias given the non-probability sample. By contrast, this is the first study conducted with this group. We have also avoided discussing prevalence and, instead, focused on the relationship between MHL and MHS, which may be generalisable. We also noted that some provinces, mainly in remote areas, had fewer participants than urban areas; this was mainly due to limited access of WiFi internet in remote areas. Due to such disproportionate sample size, this might not have yielded an accurate representation of the views of officers in these remote areas regarding their MHL and MHS. Also, we found that there was gender imbalance because the number of participating males was higher (62%) compared with women (38%). As such, future studies might want to explore the same aims and objectives using a stratified sampling method.

### Implications

Taken together, in the first study of this kind, we show that MHL is associated with MHS in officers in a prison population. We also show that MHL can be affected by various practices including training, age and possibly experience on the job. These results are important for other similar institutions in LMICs. Further research should explore which training interventions can improve MHL and reduce MHS. Perhaps healthcare providers and correctional officers working with individuals incarcerated with mental illness in Zimbabwe should be offered the MHFA, which was highlighted by previous research as being effective in increasing MHL.^35^

### Implications for non-significant findings

Based on our findings, we noted that daily contact with people incarcerated with mental illness did not reduce stigma. This could be due to adversarial relationships between correctional officers and individuals in prison with mental illness. As such, this could lead to correctional officers using punitive measures or discrimination that will result in these individuals (prisoners) becoming socially withdrawn from others, thus affecting their quality of life or leading to them not seeking treatment.^52,53^ These problems may possibly be exacerbated in a low-resource setting. However, further research is needed in this area.

Regarding the quality of the training provided to the ZPCS, we found that being trained in mental health awareness did not reduce stigma. One caveat is that we do not have information on the extent of the training provided, and it is possible that training targeted towards specific areas could influence both MHL and MHS.

## Data Availability

The data that support the findings of this study are available from the corresponding author, D.C., upon reasonable request.
